# The roles of stress, illness management, and goal setting on the association between personal and clinical recovery – a prospective study

**DOI:** 10.1186/s12888-025-07291-4

**Published:** 2025-09-02

**Authors:** Regina Skar-Fröding, Hanne Clausen, Anne Marciuch, Torleif Ruud, Jurate Šaltyte Benth, Mina Veland, Kristin S. Heiervang

**Affiliations:** 1 R&D Department, Division of Mental Health Services, Akershus University Hospital, P.O. box 1000, Lørenskog, 1478 Norway; 2 Norwegian National Advisory Unit on Concurrent Substance Abuse and Mental Health Disorders and Mental Health Division, Innlandet Hospital Trust, Brumunddal, Norway; 3 Department of Addiction, Akershus University Hospital, Lørenskog, Norway; 4 Institute of Clinical Medicine, University of Oslo, Oslo, Norway; 5 Institute of Clinical Medicine, Campus Ahus, University of Oslo, P.O.Box 1171, Blindern, 0318 Norway; 6 Health Services Research Unit, Akershus University Hospital, P.O.Box 1000, Lørenskog, 1478 Norway

**Keywords:** Clinical recovery, Personal recovery, Stress and illness management, Goal setting

## Abstract

**Background:**

Investigating underlying working mechanisms involved in the relationship between personal and clinical recovery is important for enhancing mental health treatment. This prospective study investigated the impact of two anticipated moderators, 1) receiving help with managing stress and illness, and 2) receiving help with setting and achieving goals, on the association between personal and clinical recovery at baseline and 18-month follow-up among service users with psychosis.

**Methods:**

Data were collected from service users with psychosis and their clinicians from 32 clinical sites across Norway at baseline (*n* = 318) and after 18 months (*n* = 121). Personal recovery was measured using the Questionnaire about the Process of Recovery. Linear mixed models were estimated to assess the impact of the two moderators on the association between personal and clinical recovery.

**Results:**

Service users with higher level of symptoms at baseline who stated that they received help with setting and achieving goals showed greater personal recovery over 18 months compared to those who stated that they did not receive this help. Service users with low level of functioning who stated that they received help with managing stress and illness or setting and achieving goals experienced higher levels of personal recovery than did those who stated that they did not receive this help.

**Conclusion:**

Receiving help with managing stress and illness and receiving help with setting and achieving goals, have an impact on personal recovery for service users with low levels of functioning and/or higher levels of symptoms. It might be beneficial to incorporate these aspects into treatments aimed at increasing personal recovery.

## Background

In recent decades, the relationship between subjective experiences of recovery, a concept known as personal recovery [1, 2], and clinical symptoms and psychosocial functioning, often referred to as clinical recovery [3, 4], has been a topic of interest. This research originates from studies on severe mental illness, highlighting the importance of understanding how these individuals navigate their recovery journeys [5]. The personal recovery concept refers to changes in ones attitude to life and the illness with emphasis on hope and the establishment of a meaningful life [2]. It has been framed as a unique, subjective process defined and assessed by the individual themselves [6]. Clinical recovery, on the other hand, has traditionally been the main focus of mental health services, emphasizing symptom reduction and increased functioning [7]. Others [8] have distinguished between symptomatic recovery, functional recovery, and personal recovery, indicating that these represent distinct domains of recovery. Studies have shown that recovery in symptoms does not necessarily go along with increased functioning [9] and that a large proportion of patients remain with poor functioning, even though their symptoms are in remission [10–12]. A growing body of research has shown that personal and clinical recovery often are related, that is, higher levels of experienced personal recovery are associated with higher levels of clinical recovery [13–15].

Nowadays, there is an increased focus on investigating the underlying working mechanisms and pathways involved in the relationship between personal recovery and clinical recovery [8, 13, 16–18]. A recent study found that the association between personal and clinical recovery among patients with schizophrenia was fully accounted for by the effects of disability and quality of life [16]. The authors proposed that better clinical recovery leads to fewer symptoms and less functional disability, which in turn, lead to a higher quality of life and thus increased personal recovery [16]. Another study on the underlying mechanisms involved in the relationship between personal and clinical recovery found that the cognitive interpretations and emotional components of psychotic experiences appear to interact with and predict functional and personal recovery, leading the authors to suggest that interventions focusing on cognitive interpretations of psychotic experiences and negative affect could influence multiple forms of recovery [8]. It has also been suggested that studies on the complex processes involved in the relationship between personal and clinical recovery should include concepts such as social support, coping, stigma, and self-efficacy because these themes have shown to be associated with personal recovery [19]. A study originating from a recent randomized controlled trial (RCT) on Illness Management and Recovery (IMR) investigated different components of IMR, such as insight, medication adherence, addiction, coping and social support, as determinants of clinical, functional and personal recovery. Among all components, only client-rated self-efficacy for coping with challenges and threats (coping) was associated with all three types of recovery (clinical, functional and personal recovery). The direct associations between coping and functional and personal recovery were stronger than the indirect associations via clinical recovery [20]. Originating from the same RCT study, another paper using mediation analysis to analyze the association between changes over time in illness self-management skills and personal recovery in patients with severe mental illnesses found that improvement in overall illness management was directly associated with improvements in personal recovery, and indirectly through improvements in clinical recovery and functional recovery [21]. Finally, a systematic review found that factors associated with personal recovery concepts such as empowerment, hope, and meaning were more strongly linked to personal recovery than clinical symptoms and psychosocial functioning. The authors suggest that to enhance personal recovery, treatments should focus on the elements of personal recovery itself, and that future research should focus on the interaction between how personal recovery elements and changes in symptoms influence each other over time as a result of treatment [22].

Given the importance of investigating working mechanisms and effective treatments involved in the relationship between personal and clinical recovery [22], the present study aimed to investigate whether two interventions, 1) receiving help with managing stress and illness, and 2) receiving help with setting and achieving goals, affected this relationship in some way. Receiving help with managing stress and illness is an aspect of treatment involved in both traditional clinical treatments and some personal recovery-oriented treatments such as illness management and recovery (IMR) [23, 24]. A randomized controlled study has shown that the IMR program is effective in increasing clients’ knowledge of and ability to cope with their illness and helping them work toward personal goals [25]. As research has shown that fostering hope is strongly linked to overall personal recovery [22], receiving help with setting and achieving goals was included in this study to investigate whether this affected the relationship between personal and clinical recovery (i.e., clinical symptoms and level of functioning).

The following research questions were raised: a) Does receiving help with managing stress and illness affect the relationship between personal recovery and clinical symptoms?; b) Does receiving help with managing stress and illness affect the relationship between personal recovery and level of functioning?; c) Does receiving help with setting and achieving goals affect the relationship between personal recovery and clinical symptoms?; and d) Does receiving help with setting and achieving goals affect the relationship between personal recovery and level of functioning?

## Methods

### Design

This prospective study used data from baseline and 18-month follow-up from the Norwegian research project A Pairwise Randomized Study on Implementation of Guidelines and Evidence-based Treatments of Psychoses (ClinicalTrials NCT03271242, registration date august 30th 2017). This study was approved by the Regional Committee for Medical and Health Research Ethics (REK Sørøst B 2015/2169) and the data protection officers at the participating health thrusts and followed the principles of the Declaration of Helsinki.

### Sample and setting

A total of 325 service users and their clinicians from 32 clinical units completed questionnaires at baseline. Due to missing data on the Questionnaire about the Process of Recovery (QPR) outcome measure [26], the final sample was reduced to 318. At 18-month follow-up, the sample included 121 service users from 26 clinical units.

The study participants were service users with psychosis receiving specialized mental health services who were recruited from six different health thrusts in Norway, including three university hospitals. Participants were recruited from hospital departments and community mental health centers, comprising outpatient clinics, day units, mobile teams, and local inpatient wards. The inclusion criteria were that the service user had been diagnosed with and/or treated for psychosis at a service unit specialized in psychosis and was at least 16 years of age. The only exclusion criterion was the inability to understand and respond to the questionnaires in Norwegian.

### Procedure

Clinicians from the participating mental health units recruited eligible service users who were either already in contact with the clinic during the study period or had recently been referred to the clinic. The clinicians conducted clinical assessments, and questionnaires were given to the service users by either the secretary or another staff member at the unit. Service users completed the questionnaires at the clinic, where they were provided with a designated place to sit, or at home. After completing the questionnaires, they sealed them in an envelope and returned them to the clinic. The recruitment phase spanned from June 2016 to March 2017, and only participants who provided written informed consent were included in the study.

An expert by experience has actively contributed to the planning, selection of relevant covariates, interpretation of results, and discussions presented in this paper.

### Measures

#### Outcome measure

The QPR is a self-report questionnaire that assesses patients’ levels of personal recovery through 15 items, which are rated on a five-point Likert scale (0, “disagree strongly”; 1, “disagree”; 2, “neither agree nor disagree”; 3, “agree”; and 4, “agree strongly”). The total sum score ranges from 0 (low recovery) to 60 (high recovery). The QPR is one of the personal recovery measures that has been considered to most closely map to the CHIME (Connectedness, Hope, Identity, Meaning, Empowerment) domains of recovery [27]. It includes items such as “I feel better about myself”, “I feel that my life as a purpose”, and “I can take charge of my life”. The QPR was developed through collaboration between clinicians and service user researchers and has demonstrated adequate psychometric properties [28]. Psychometric evaluation of the QPR in the current sample showed a one-factor solution with high internal consistency (Cronbach’s alpha 0.91).

#### Covariates

As previous literature has shown a discrepancy between the level of clinical symptom and functioning among people with psychosis [8, 11], we included these as separate factors of clinical recovery in their relation to personal recovery.

##### Clinical symptoms

The clinical symptoms variable included symptoms of depression and psychosis, as a recent study has proposed that the concept of clinical recovery should involve both psychotic and affective symptoms [3]. In the present study, a composite score from two subscales (Depression/functioning and Hallucinations and Delusions) from the Health of the Nation Outcome scale (HoNOS) was used/included [29]. In the HoNOS scale, each subscale is rated from 0 to 4, so the composite variable score in this study had a range from 0 to 8, with higher scores indicating more problems or more severe symptoms.

##### Level of functioning

The level of functioning variable was based on three dichotomous variables, ultimately giving a score between 0 and 3 based on how many “yes” the participant were assigned on each dichotomous variable, with higher scores indicating a higher level of functioning. The dichotomous variables included were based on a recent definition of functional recovery [3], and incorporated independent living (yes/no), occupational function (yes/no), and having one or more close friends (yes/no). The participants were considered to be living independently if they resided in an unsupervised home, including not living with their parents. In the occupational function variable, participants were assigned “yes” if they were in either full- or part-time regular employment or if they were full- or part-time students. “Have one or more close/confident friends“ was based on an item from the Practical and Social Function Scale [30]. Participants were included as “yes” if the clinician rated the item as “completely correct” or “correct to a large degree”, and “no” if the item was rated as “partly correct”, “correct to a small degree”, or “completely incorrect”.

#### Moderators

Two potential moderators were included. These variables were based on two self-reported items covering interventions that the service users stated that they had received for the last 6 months prior to inclusion. Participants rated the following two statements: “I have received good help in coping with stress and illness” and “I have received good help with setting and achieving goals” on a graded scale (“disagree”, “agree to a small extent “, “partly agree”, “largely agree”, and “totally agree”). Participants were included as “yes” if they rated the item as “largely agree” or “totally agree”, and “no” if the item was rated as “partly agree”, “agree to a small extent”, or “disagree”.

### Analysis

Participant characteristics were presented as means and standard deviations (SDs), and frequencies and percentages, as appropriate. Correlation analysis between clinical symptoms and level of functioning has been reported in a previous paper [31]. The present study assessed differences between the drop-out sample and the follow-up sample for “receiving help with managing stress and illness” and “receiving help with setting and achieving goals” by an independent-samples *t*-test or a χ^2^-test.

To assess whether the association between change in the outcome variable, QPR, and change in the covariates of clinical symptoms and level of functioning, was modified by the covariates “Receiving help with managing stress and illness” and “Receiving help with setting and achieving goals” assessed at baseline, eight linear mixed models with random effects for participants were estimated. Model 1 and Model 3 included fixed effects for a time dummy (baseline and follow-up), covariate clinical symptoms measured simultaneously with outcome, and an interaction between time and covariate. The modifiers “Receiving help with managing stress and illness” and “Receiving help with setting and achieving goals” were included in Models 1 and 3, respectively, with all two- and three-way interactions. Models 2 and 4 were the same as Models 1 and 3, respectively, but with clinical symptoms measured longitudinally substituted by clinical symptoms measured at baseline. Models 5–8 were estimated by substituting the clinical symptoms in Models 1–4 with level of functioning.

To ease the interpretation of interaction terms, post hoc analyses were performed. Results were presented as mean QPR values at each time point within each group and illustrated graphically. Relevant comparisons were presented as mean differences with corresponding 95% confidence intervals and *p*-values. The results with *p*-values < 0.05 were considered statistically significant. All statistical analyses were performed in STATA v.18.

## Results

The sociodemographic and clinical characteristics of the participants are shown in Table 1.

**Table 1:** Clinical and sociodemographic characteristics of the participants at baseline (*n* = 318) and 18-month follow-up (*n* = 121)

Clinical and sociodemographic characteristics	Baseline	Follow-up
Age		
* n*	307	118
Min–max	16–77	19–77
Mean (SD)	40.1 (12.7)	42.1 (12.2)
Gender		
* n*	317	119
Female	130 (41%)	60 (50%)
Male	187 (59%)	59 (50%)
Diagnosis		
* n*	300	112
Schizophrenia (F20–20.9)	160 (53%)	57 (51%)
Paranoid psychosis (F22–22.9)	21 (7%)	8 (7%)
Schizoaffective disorders (F25–25.9)	60 (20%)	25 (22%)
Acute polymorph psychosis (F23–23.9)	10 (3%)	6 (5%)
Schizotypal Dirsorder (F21), Other psychotic disorder not due to a substance or known physiological condition (F28), Unspecified psychosis not due to a substance or known physiological condition (F29)	22 (7%)	4 (4%)
Other	27 (9%)	12 (11%)
Questionnaire about the Process of Recovery		
* n*	318	121
Min–max	5–60	0–60
Mean (SD)	40.8 (10.3)	40.9 (10.7)
Clinical symptoms		
* n*	271	88
Min–max	0–7	0–6
Mean (SD)	2.0 (1.7)	1.8 (1.5)
Level of functioning		
* n*	304	93
0	38 (12.5)	9 (9.7)
1	109 (35.9)	46 (49.5)
2	134 (44.1)	33 (35.5)
3	23 (7.6)	5 (5.4)
Receiving help with managing stress and illness		
* n*	324	
Yes	164 (50.6)	
No	160 (49.4)	
Receiving help with setting and achieving goals		
* n*	323	
Yes	164 (50.8)	
No	159 (49.2)	

*SD* standard deviation

### Drop-out analysis

Results from the drop-out analysis have been described in a previous paper [31], showing that the drop-out sample (*n* = 198) exhibited a significantly larger proportion of males (*p* = 0.01) and had a significantly lower mean score on the QPR (*p* = 0.011) compared with the follow-up sample (*n* = 120). However, even though significant, the difference in QPR score was small with the drop-out sample having a mean score of 40 (SD = 11.3) and the follow-up sample having a mean score of 42 (SD = 8.4). The present study found no significant differences in “receiving help with managing stress and illness” or “receiving help with setting and achieving goals” between the drop-out and follow-up samples. There were also no significant differences between the drop-out sample and the follow-up sample regarding clinical symptoms and level of functioning.

### Correlation between clinical symptoms and level of functioning

As previously reported [31], a weak negative correlation was observed between the level of symptoms and the level of functioning at baseline (–0.24), and at follow-up (–0.07).

### The role of receiving help with managing stress and illness as effect modifier on the associations between clinical symptoms and personal recovery

Table 2 presents linear mixed model results for associations between personal recovery (QPR) at baseline (*n* = 266) and 18-month follow-up (*n* = 82) and clinical symptoms measured simultaneously, with receiving help with managing stress and illness included as effect modifier (Model 1), and for associations between personal recovery (QPR) at baseline (*n* = 266) and 18-month follow-up (*n* = 101) and clinical symptoms at baseline with receiving help with managing stress and illness included as effect modifier (Model 2).

**Table 2:** Managing stress and illness as effect modifier on the association between clinical symptoms and personal recovery

Model 1	RC (SE)	*p*-value
Intercept	41.83 (1.38)	<0.001
Time (reference: BL)	2.93 (2.10)	0.164
Clinical symptoms	–1.65 (0.54)	**0.002**
Time × clinical symptoms	–2.39 (1.21)	**0.049**
Stress and illness management (reference: no)	3.52 (1.74)	**0.043**
Time × stress and illness management	–1.59 (2.75)	0.563
Clinical symptoms × stress and illness management	0.27 (0.69)	0.696
Time × clinical symptoms × stress and illness management	0.92 (1.54)	0.549
Model 2	RC (SE)	*p*-value
Intercept	42.03 (1.41)	<0.001
Time (reference: BL)	1.43 (2.15)	0.505
BL clinical symptoms	–1.74 (0.56)	**0.002**
Time × BL clinical symptoms	–1.00 (0.94)	0.285
Stress and illness management (reference: no)	4.02 (1.78)	**0.024**
Time × stress and illness management	–0.66 (2.49)	0.790
BL clinical symptoms × stress and illness management	0.03 (0.71)	0.971
Time × BL clinical symptoms × stress and illness management	0.29 (1.09)	0.789

*BL* baseline, *RC* regression coefficient, *SE* standard error

Boldface are significant at 0.05 level

#### Clinical symptoms and personal recovery assessed simultaneously at baseline and 18-month follow-up

None of the two- or three-way interactions were significant, implying that receiving help with managing stress and illness did not affect the overall associations between clinical symptoms and QPR (Table 2, Model 1).

#### Clinical symptoms at baseline, and change in and level of personal recovery at 18-month follow-up

None of the two- or three-way interactions for receiving help with managing stress and illness were significant, implying that it did not affect the associations between clinical symptoms at baseline and QPR at 18-month follow-up (Table 2, Model 2).

### The role of receiving help with managing stress and illness as effect modifier in the relationship between level of functioning and personal recovery

Table 3 presents linear mixed model results for associations between level of functioning and personal recovery (QPR) at baseline (*n* = 296) and 18-month follow-up (*n* = 86) with receiving help with managing stress and illness as the effect modifier (Model 3), and linear mixed model results for associations between level of functioning at baseline (*n* = 296) and personal recovery (QPR) at 18-month follow-up (*n* = 109) with receiving help with managing stress and illness as the effect modifier (Model 4).

**Table 3:** Managing stress and illness as the effect modifier on the association between clinical symptoms and personal recovery

Model 3	RC (SE)	*p*-value
Intercept	37.60 (1.64)	<0.001
Time (reference: BL)	–0.16 (2.83)	0.954
Level of functioning (reference: 0)		
1	–1.16 (1.98)	0.557
2	3.45 (2.03)	0.089
3	0.92 (4.05)	0.819
Time × level of functioning		
1	0.61 (3.56)	0.864
2	–3.40 (4.11)	0.408
3	7.18 (3.19)	**0.024**
Stress and illness management (reference: yes)	4.96 (1.85)	**0.007**
Time × stress and illness management	–11.71 (5.25)	**0.026**
Level of functioning × stress and illness management		
1	–0.60 (2.43)	0.806
2	–1.86 (2.42)	0.442
3	1.83 (4.92)	0.710
Time × level of functioning × stress and illness management		
1	13.21 (5.84)	**0.024**
2	15.30 (6.60)	**0.021**
3	10.41 (5.88)	0.077
Model 4	RC (SE)	*p*-value
Intercept	36.40 (1.77)	<0.001
Time (reference: BL)	–3.07 (1.79)	0.087
BL level of functioning (reference: 0)		
1	0.29 (2.25)	0.896
2	4.75 (2.22)	**0.033**
3	3.27 (4.32)	0.449
Time × BL level of functioning		
1	2.62 (3.25)	0.419
2	–0.12 (3.06)	0.969
3	9.51 (2.47)	**<0.001**
Stress and illness management (reference: yes)	6.32 (2.09)	**0.002**
Time × stress and illness management	2.76 (3.89)	0.477
BL level of functioning × stress and illness management		
1	–2.88 (2.86)	0.314
2	–3.01 (2.74)	0.271
3	–0.39 (5.42)	0.943
Time × BL level of functioning × stress and illness management		
1	–0.93 (5.02)	0.854
2	–0.68 (4.88)	0.889
3	–9.80 (4.81)	**0.042**

*BL* baseline, *RC* regression coefficient, *SE* standard error

Boldface are significant at 0.05 level

#### Level of functioning and personal recovery assessed simultaneously at baseline and 18-month follow-up

**Fig. 1:**
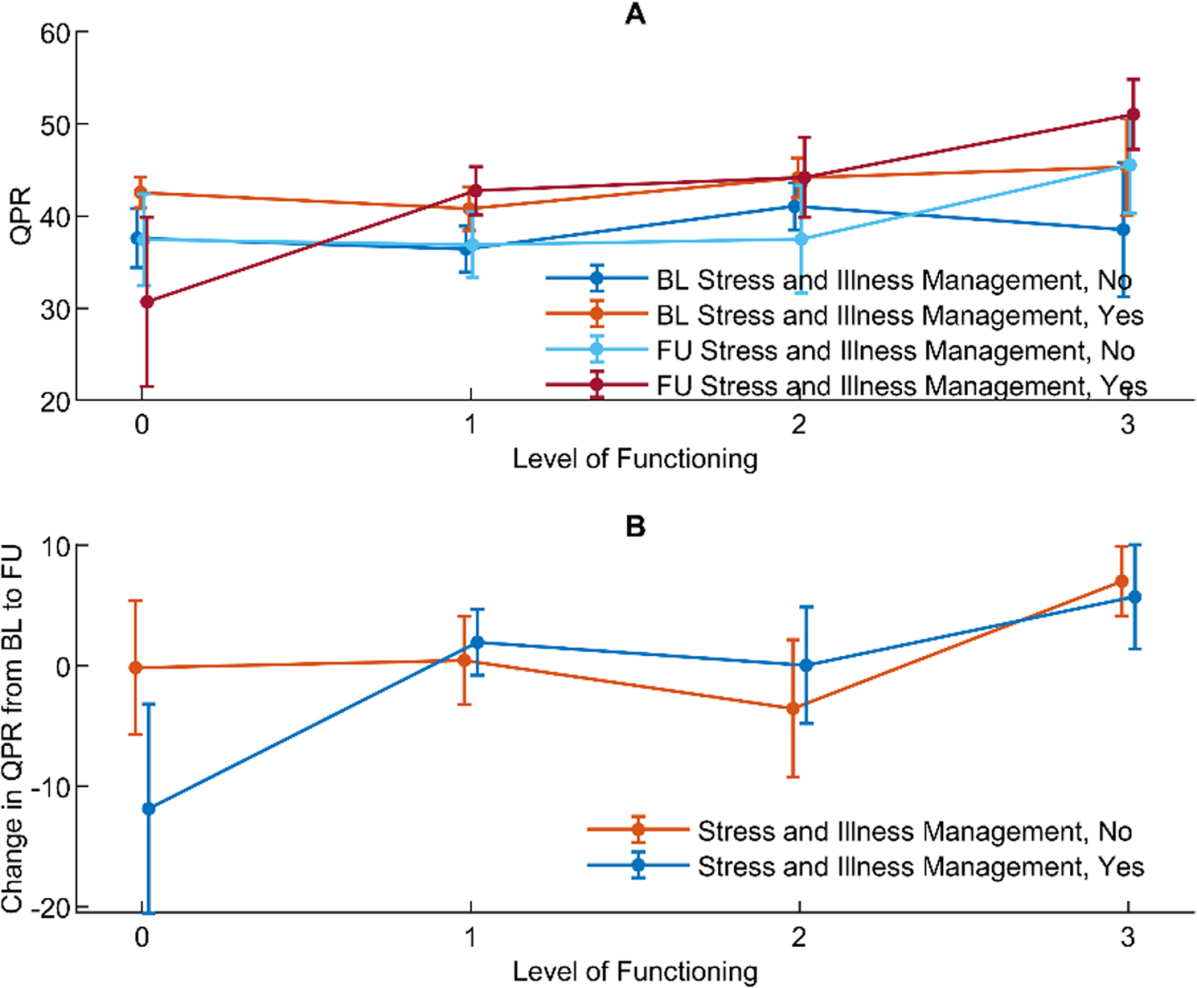
Associations between level of functioning and QPR with managing stress and illness as effect modifier

The presence of significant interactions in Model 3 (Table 3) indicates that receiving help with managing stress and illness affected the association between level of functioning and QPR. At baseline, QPR scores were significantly lower for those not receiving help with managing stress and illness than for those receiving help for level of functioning-0 (*p* = 0.007) and level of functioning-1 (*p* = 0.013). At 18-month follow-up, QPR scores were significantly lower among those not receiving help with managing stress and illness than for those receiving help for level of functioning-1 (*p* = 0.009) (Fig. 1A).

Regarding change in QPR from baseline to follow-up, the differences between those receiving and not receiving help with managing stress and illness were significant between level of functioning-0 and level of functioning-1 (*p* = 0.024, three-way interaction), and between level of functioning-0 and level of functioning-2 (*p* = 0.021, three-way interaction) (Fig. 1B).

#### Level of functioning at baseline, and change in and level of personal recovery at 18-month follow-up

A significant interaction between time, level of functioning at baseline, and receiving help with managing stress and illness indicates that receiving help with managing stress and illness might have affected the association between level of functioning at baseline and QPR (Table 3, Model 4).

**Fig. 2:**
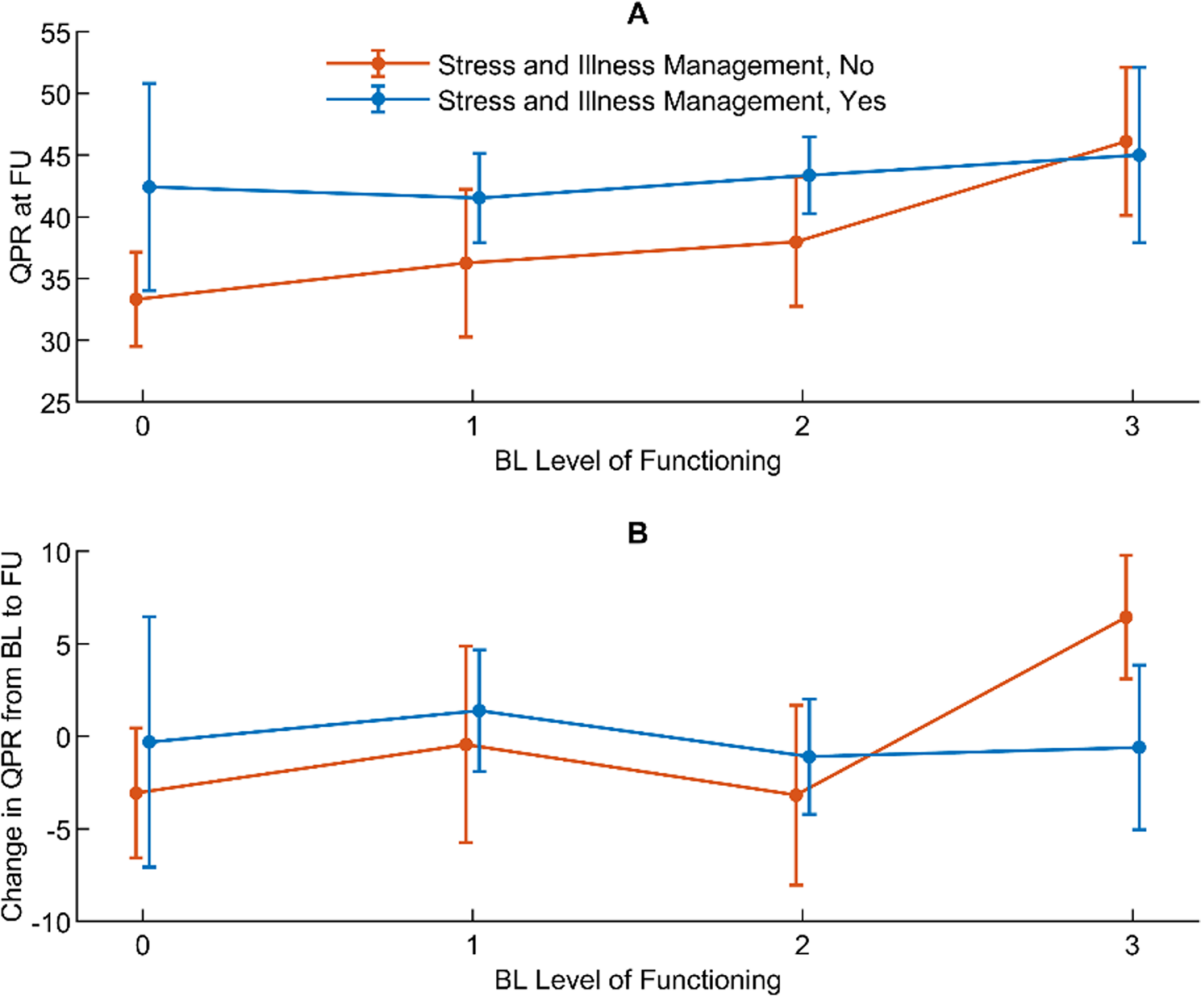
Associations between baseline level of functioning and QPR with managing stress and illness as effect modifier

At follow-up, no significant differences were observed between those receiving and not receiving help with managing stress and illness in the overall association between level of functioning at baseline and QPR (Fig. 2A). However, differences in change in QPR from baseline to follow-up between those receiving and not receiving help with managing stress and illness were significant between level of functioning-0 and level of functioning-2 (*p* = 0.042, three-way interaction), level of functioning-1 and level of functioning-3 (*p* = 0.037, post hoc analysis), and level of functioning-2 and level of functioning-3 (*p* = 0.026, post hoc analysis) (Fig. 2B).

### The role of receiving help with setting and achieving goals as effect modifier on associations between clinical symptoms and personal recovery

Table 4 presents linear mixed model results for associations between clinical symptoms and personal recovery (QPR) at baseline (*n* = 266) and at 18-month follow-up (*n* = 82) with receiving help with setting and achieving goals as effect modifier at baseline (Model 5), and linear mixed model results for associations between clinical symptoms at baseline (*n* = 266) and personal recovery (QPR) at 18-month follow-up (*n* = 101) with receiving help with setting and achieving goals as effect modifier (Model 6).

**Table 4:** Setting and achieving goals as effect modifier on the association between clinical symptoms and personal recovery

Model 5	RC (SE)	*p*-value
Intercept Time (reference: BL) Clinical symptoms Time × clinical symptoms Setting and achieving goals (reference: no) Time × setting and achieving goals Clinical symptoms × setting and achieving goals Time × clinical symptoms × setting and achieving goals	41.17 (1.43) 2.19 (2.19) –1.22 (0.55) –2.47 (1.23) 4.23 (1.78) –0.71 (2.77) –0.33 (0.70) 1.37 (1.48)	< 0.001 0.317 **0.026** **0.044** **0.017** 0.797 0.632 0.353
Model 6	RC (SE)	*p*-value
Intercept Time (reference: BL) BL clinical symptoms Time × BL clinical symptoms Setting and achieving goals (reference: no) Time × setting and achieving goals BL clinical symptoms × setting and achieving goals Time × BL clinical symptoms × setting and achieving goals	41.57 (1.48) 3.37 (1.73) –1.39 (0.57) –2.28 (0.88) 4.37 (1.83) –2.99 (2.21) –0.43 (0.73) 2.24 (0.99)	< 0.001 0.052 **0.015** **0.009** **0.017** 0.176 0.556 **0.024**

*BL* baseline, *RC* regression coefficient, *SE* standard error

Boldface are significant at 0.05 level

#### Clinical symptoms and personal recovery assessed simultaneously at baseline and 18-month follow-up

None of the two- or three-way interactions in Model 5 were significant, implying that receiving help with setting and achieving goals did not affect the overall associations between clinical symptoms and QPR (Table 4, Model 5).

#### Clinical symptoms at baseline and change in and level of personal recovery at 18-month follow-up

**Fig. 3:**
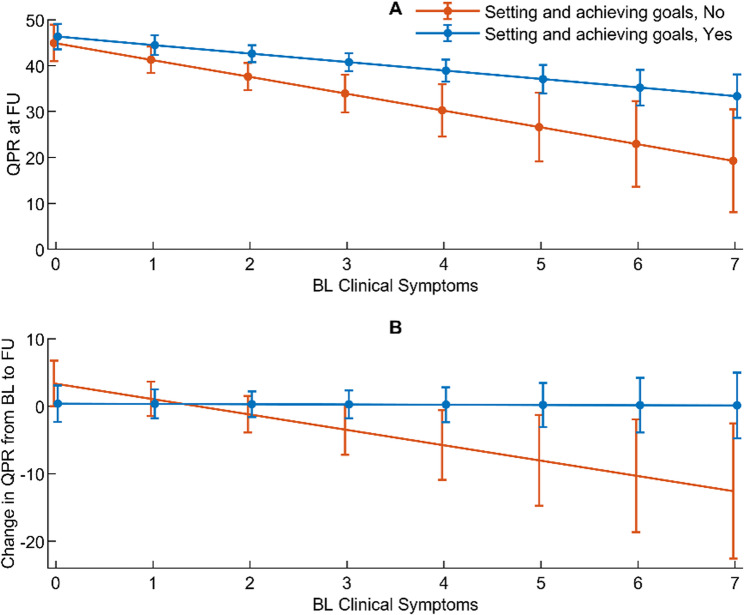
Associations between baseline clinical symptoms and QPR with setting and achieving goals as effect modifier

The three-way interaction between time, receiving help with setting and achieving goals, and clinical symptoms at baseline was significant (*p* = 0.024), which indicates that receiving help with setting and achieving goals affects the association between clinical symptoms at baseline and the change in QPR from baseline to 18-month follow-up (Table 4, Model 6). The association between clinical symptoms at baseline and QPR at 18-month follow-up was significant among those both receiving and not receiving help with setting and achieving goals, but was significantly stronger among those not receiving help with setting and achieving goals for baseline clinical symptom values of ≥ 2 (Fig. 3A).

Further, change in QPR from baseline to follow-up was significant for those not receiving help with setting and achieving goals for baseline clinical symptoms values of ≥ 4, but not for those receiving help with setting and achieving goals (Fig. 3B). The difference between those receiving and not receiving help with setting and achieving goals regarding the association between clinical symptoms at baseline and change in QPR was significant for clinical symptom values of ≥ 4.

### The role of receiving help with setting and achieving goals as effect modifier on associations between level of functioning and personal recovery

Table 5 presents linear mixed model results for associations between level of functioning and personal recovery (QPR) at baseline (*n* = 296) and 18-month follow-up (*n* = 86), with receiving help with setting and achieving goals as effect modifier (Model 7), and linear mixed model results for associations between level of functioning at baseline (*n* = 296) and personal recovery (QPR) at 18-month follow-up (*n* = 109), with receiving help with setting and achieving goals as the effect modifier (Model 8).

**Table 5:** Setting and achieving goals as effect modifier on associations between clinical symptoms and personal recovery

Model 7	RC (SE)	*p*-value
Intercept	37.65 (1.43)	<0.001
Time (reference: BL)	–10.03 (4.61)	**0.030**
Level of functioning (reference: 0)		
1	–0.98 (1.84)	0.592
2	2.52 (1.90)	0.185
3	1.85 (4.30)	0.666
Time × level of functioning		
1	9.99 (4.88)	**0.041**
2	7.66 (5.70)	0.179
3	16.70 (4.98)	**0.001**
Setting and achieving goals (reference: yes)	4.61 (1.94)	**0.018**
Time × setting and achieving goals	8.71 (4.99)	0.081
Level of functioning × setting and achieving goals		
1	–0.95 (2.55)	0.708
2	0.33 (2.52)	0.896
3	–0.11 (5.19)	0.983
Time × level of functioning × setting and achieving goals		
1	–5.75 (5.56)	0.301
2	–7.76 (6.51)	0.233
3	–9.29 (5.58)	0.096
Model 8	RC (SE)	*p*-value
Intercept	35.58 (1.50)	<0.001
Time (reference: BL)	–4.27 (1.74)	**0.014**
BL level of functioning (reference: 0)		
1	–1.03 (2.04)	0.614
2	2.67 (2.04)	0.190
3	3.14 (4.54)	0.488
Time × BL level of functioning		
1	2.95 (3.30)	0.371
2	3.23 (2.84)	0.256
3	9.55 (2.68)	**<0.001**
Setting and achieving goals (reference: yes)	4.92 (2.22)	**0.027**
Time × setting and achieving goals	5.47 (3.49)	0.117
BL level of functioning × setting and achieving goals		
1	–1.39 (2.97)	0.638
2	0.03 (2.84)	0.993
3	–1.64 (5.69)	0.773
Time × BL level of functioning × setting and achieving goals		
1	–1.57 (4.62)	0.735
2	–6.38 (4.47)	0.154
3	–9.97 (4.73)	**0.035**

*BL* baseline, *RC* regression coefficient, *SE* standard error

Boldface are significant at 0.05 level

#### Level of functioning and personal recovery assessed simultaneously at baseline and 18-month follow-up

Interactions between receiving help with setting and achieving goals and other variables in the model were not significant, implying that receiving help with setting and achieving goals did not affect the overall association between level of functioning and QPR (Table 5, Model 7).

#### Level of functioning at baseline, and change in and level of personal recovery at 18-month follow-up

The interaction between time, level of functioning at baseline, and receiving help with setting and achieving goals was significant, which indicates that receiving help with setting and achieving goals might have affected the association between level of functioning at baseline and QPR (Table 5, Model 8).

**Fig. 4:**
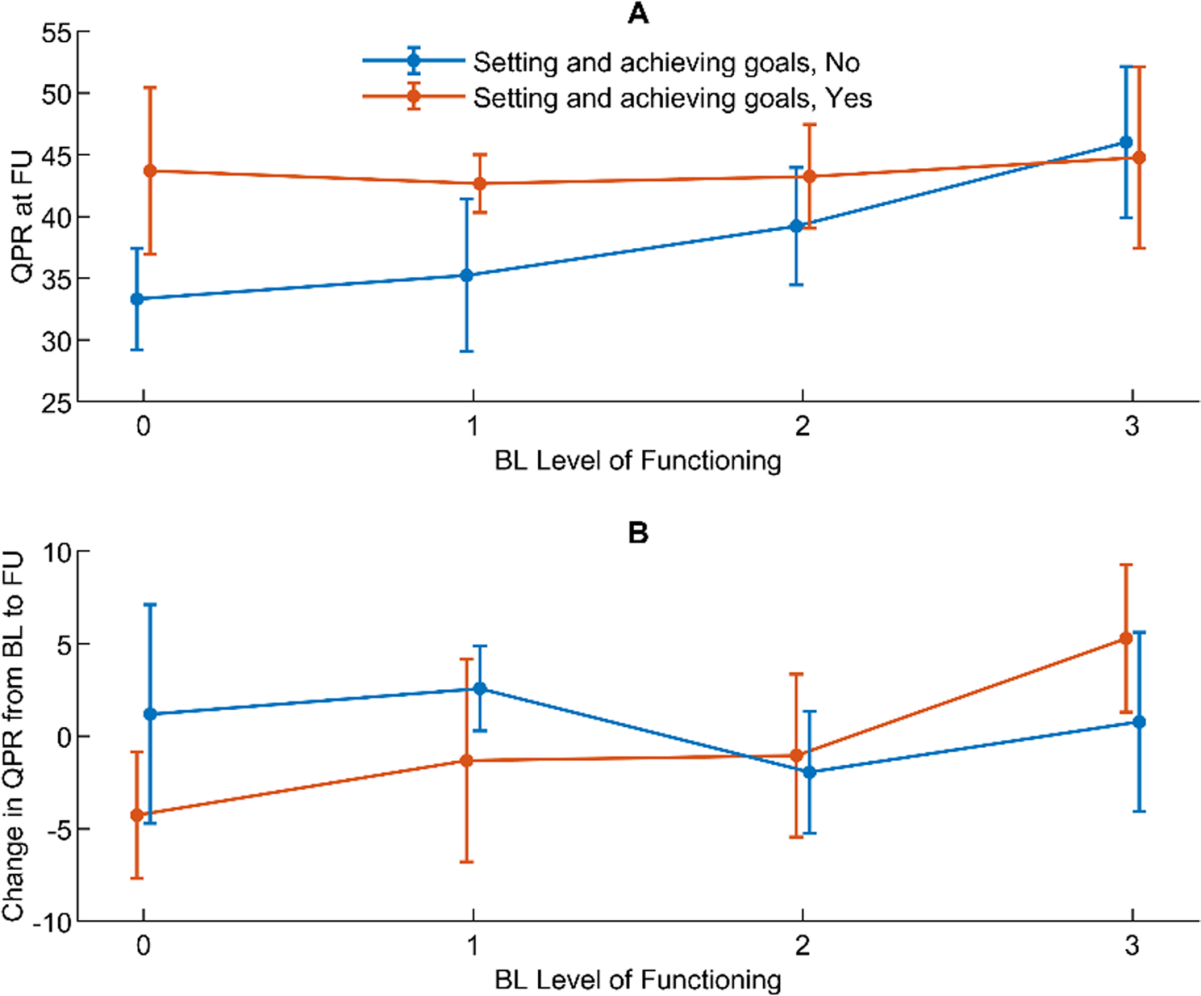
Associations between baseline level of functioning and QPR with setting and achieving goals as effect modifier

At follow-up, significant differences were seen between those receiving and not receiving help with setting and achieving goals in the association between level of functioning at baseline and QPR at 18-month follow-up for level of functioning-0 (*p* = 0.010) and level of functioning-1 (*p* = 0.028) (Fig. 4A). Differences were also observed between those receiving and not receiving help with setting and achieving goals in the association between level of functioning at baseline and change in QPR from baseline to 18-month follow-up between level of functioning-0 and level of functioning-3 (*p* = 0.035, three-way interaction) (Fig. 4B).

## Discussion

The findings of the present study highlight the positive contributions of receiving help with managing stress and illness, as well as setting and achieving goals, in supporting and maintaining personal recovery.

Although we did not find an effect of either receiving help with managing stress and illness (Table 2, Model 1) or setting and achieving goals (Table 4, Model 5) in the overall associations between clinical symptoms and personal recovery, we did find that service users with a moderate to high level of symptoms at baseline who received help with setting and achieving goals showed greater personal recovery over 18 months (Fig. 3A and B) than did those who did not receive such help. This indicates that for service users with higher levels of symptoms, receiving help with setting and achieving goals appears to be meaningful for enhancing and sustaining their personal recovery over time. Although only speculating, this finding may be due to the greater challenges faced by individuals with more pronounced symptoms in their daily lives. Support in setting and achieving goals might provide a framework for these individuals, helping them to gain a sense of hope and a feeling of accomplishment. In contrast, those with milder symptoms might already possess a higher degree of self-efficacy, making the impact of additional goal-setting support less pronounced.

We found that both receiving help with managing stress and illness and setting and achieving goals affected the relationship between level of functioning and personal recovery in some ways. For service users with lower levels of functioning, receiving help with managing stress and illness contributed significantly to personal recovery, as indicated by higher levels of personal recovery at both baseline and follow-up (Fig. 1A). This indicates that such support is particularly beneficial for service users with lower levels of functioning to strengthen their personal recovery. For example, mental health professionals can assist individuals by helping them understand the relationship between stress and worsening of symptoms, developing personalized coping plans to manage stress and illness in their daily lives. This can strengthen an individual’s resilience and reduce the likelihood of increased symptoms. However, receiving help with managing stress and illness did not affect the longitudinal relationship between level of functioning at baseline and personal recovery at follow-up (Fig. 2A and B).

Although receiving help with setting and achieving goals did not affect the overall association between level of function and personal recovery (Table 5, Model 7), we found that those with lower levels of functioning at baseline who received help with setting and achieving goals had a significantly higher level of personal recovery after 18 months (Fig. 4A) than did those who did not receive such help. This finding indicates that for those with low levels of functioning, receiving help with setting and achieving goals is important for maintaining and strengthen their personal recovery over time. Mental health professionals can play a key role in helping individuals identify personally meaningful goals, break them down into manageable steps and provide support and feedback for attaining those goals. In the field of personal recovery, a distinction is often made between recovery as a process and recovery as an outcome. Personal recovery is frequently cited as an ongoing process, while clinical recovery is viewed as an outcome with an endpoint [32]. Goal setting typically reflects an outcome-oriented perspective, focused on goal attainment. However, since personal recovery is a subjective process of building meaning, identity, and hope [33], the act of setting personally defined and meaningful goals can serve both as a tool for measurable progress and as a means to foster the recovery journey.

The overall pattern of these findings shows that for those with lower levels of clinical recovery (high symptoms and low level of functioning), receiving help with managing stress and illness and setting and achieving goals has a positive impact on enhancing personal recovery. These findings have clinical implications. They show that treatment aspects involved in both traditional clinical treatments and some personal recovery-oriented treatments, such as receiving help with managing stress and illness, and more specific personal recovery-oriented treatment aspects, such as receiving help with setting and achieving goals, are important for maintaining and increasing personal recovery. This dual approach of combining traditional clinical recovery support with personal recovery-oriented interventions can be especially important in helping individuals with significant challenges work toward a more fulfilling life. Receiving help with setting and achieving goals seems to be an intervention of special importance as it had a significant impact for both those with high symptoms and a low level of functioning. Aligning treatment with service users’ personal goals is a core component of personal recovery, as it allows service users to express what is truly important to them [34, 35] and has been incorporated into several recovery treatment interventions [24, 36, 37]. Setting concrete goals and working toward them outside the therapy room has the potential to influence the everyday life, contributing to a sense of mastery and allowing people to see progress. Working toward a concrete goal might also contribute to empowerment and reduce public stigma, which often portrays people with a mental illness as needing someone else to make decisions about their goals [38]. Finally, time also seems to be of importance, as receiving help with setting and achieving goals showed importance for enhancing the personal recovery process over time. The importance of time may reflect some underlying processes. Achieving meaningful goals often takes time, especially for individuals with mental health challenges. The longer duration might also indicate ongoing engagement with treatment, more stable therapeutic relationships, or the effects of medication stabilization for those who choose to use medication, contributing to an improved capacity to pursue goals. Future research could explore the mechanisms behind these time-dependent effects through longitudinal studies that account for treatment intensity, therapeutic alliance, and specific intervention content. This could shed light on how these factors interact over time to support recovery.

### Strengths and limitations

A major strength of this study is the rather large and diverse sample of participants with psychosis in different services settings, which allowed us to obtain real-world information that increases the generalizability of the study. However, a limitation of this study is the potential selection bias. Although the participating clinical units are considered representative of psychosis treatment in the Norwegian mental health-care system, the participants were not randomly selected. In addition, the clinicians who recruited the participants were instructed to recruit/ask all eligible service users, but we do not have information on the actual numbers of participants who were asked to participate. Hence, this sample might be a convenience sample, which limits the generalizability of the findings. We also might have lost some of the variance present because of the dichotomizing of some of the items included in the level of functioning variable and moderator variables. No formal power analysis was conducted to determine the sample size required to detect meaningful effects for this study, as it is part of a larger study. However, we carefully evaluated the number of variables we could include in the linear mixed models based on the number of participants. Finally, the drop-out analysis revealed a difference in QPR score and a higher percentage of males who discontinued participation, potentially impacting follow-up results. However, although the difference in QPR scores was statistically significant, it was small. The lack of standard administration of how participants received the interventions also affects the validity and reliability of the research findings.

## Conclusion

Overall, the results of this study indicate that receiving help with managing stress and illness and receiving help with setting and achieving goals affect the relationship between personal and clinical recovery for those with lower levels of clinical recovery. These findings suggest that receiving help with managing stress and illness and setting and achieving goals might have a significant impact on personal recovery for service users with low levels of functioning and high levels of symptoms. These treatment aspects could be beneficial to incorporate into treatments aimed at increasing personal recovery.

## Data Availability

The datasets used and/or analysed during the current study are available from the corresponding author on reasonable request.

## Abbreviations

IMR: Illness Management and Recovery
QPR: Questionnaire about the Process of Recovery
HoNOS: Health of the Nation Outcome scale
SD: Standard Deviation
BL: Baseline
RC: Regression coefficient
SE: Standard error
